# Six-Month Soft Tissues Healing after Lower Third Molar Extraction: Comparison of Two Different Access Flaps

**DOI:** 10.3390/jcm12227017

**Published:** 2023-11-09

**Authors:** Alessia Pardo, Annarita Signoriello, Martina Corrà, Vittorio Favero, Rachele De’Manzoni Casarola, Massimo Albanese

**Affiliations:** Dentistry and Maxillofacial Surgery Unit, Department of Surgery, Dentistry, Paediatrics and Gynaecology (DIPSCOMI), University of Verona, Piazzale L.A. Scuro 10, 37134 Verona, Italy; marty060999@gmail.com (M.C.); vittorio.favero@univr.it (V.F.); rachele.demanzonicasarola@univr.it (R.D.C.); massimo.albanese@univr.it (M.A.)

**Keywords:** extraction, healing, periodontal disease, third molar surgery

## Abstract

Background: As specific flap designs performed for lower third molar extractions usually influence periodontal healing of the adjacent first and second molars, this study aimed to evaluate the periodontal conditions of these sites after 6 months post-surgery. Methods: Forty patients, aged 14–30 years, were included. Surgical extraction of the lower third molar was performed through a flap with papilla detachment (a modified envelope technique with detachment of gingival papilla between the first and second molars) or a trapezoidal flap (characterized by mesial- and distal-releasing incisions). Periodontal parameters at the first and second molar sites were assessed for visible plaque index, bleeding on probing, recession, probing pocket depth, and clinical attachment loss before surgery (T0), one month (T1), and six months after extraction (T2). Results: No statistical differences were found for the plaque and bleeding indexes between the two flaps at each observation time and considering both time intervals. For recession, no statistical differences were found between the two flaps considering the final time interval. For probing pocket depth at the second molar site, both techniques registered a significant increase between T0 and T1, followed by a decrease up to T2. For clinical attachment loss, mean values assessed for the first and second molar sites demonstrated evidently increased values between T0 and T1, followed by moderate decreases up to T2. Conclusions: Considering short (T1) and mid-term (T2) follow-ups, a specific flap design does not seem to particularly influence periodontal healing six months after surgery.

## 1. Introduction

Extraction of third molars represents a frequently invasive surgical procedure for both hard and soft tissues, particularly in the lower jaw. As the design of the access flap, gingival detachment included, often implicates a considerable damage for the entire periodontium, periodontal healing seems to depend on the chosen surgical technique [1]. Additional factors influencing the quality and course of the healing process must be taken into consideration, including the patient’s age, patient’s systemic conditions and eventual medications taken, periodontal conditions of the adjacent second molar, position of the third molar and inclination of its roots, relationship between the third molar and the inferior alveolar nerve, relationship between the third molar and the second molar, and the degree of openness of the patient’s mouth [1,2,3,4,5,6].

Being a relevant violation of soft tissues almost inevitable in performing incisions for the surgical removal of third molars, investigations available in the literature [7,8] mainly focused on the assessment of the periodontal probing pocket depth (PPD) and clinical attachment loss (CAL) at a distal site of the second molar, with recurring development of periodontal pockets in this specific site [9,10]. As reported by some authors [7], recession of the gingival margin level (REC), bleeding on probing (BOP), and visible plaque index (VPI) were considered for a complete evaluation of soft tissue healing.

Although comparisons of different types of surgical access revealed conflicting outcomes regarding their precise influence on post-surgical periodontal conditions, the techniques proposed can be summed up and classified [11] as envelope flap (which provides an intrasulcular vestibular incision starting from the central vestibular of the first molar clinical crown, then continuing distally with the intrasulcular gingival incision of the second molar, and finally ending with a release incision in the distal vestibular direction [12]), triangular flap (which utilizes an additional releasing incision from the distobuccal side of the second molar [13]), trapezoidal flap (in which a mesial-releasing incision starting from the distal buccal gingival margin of the first molar is associated to the distal one to allow easier flap dissection [12]), and para-marginal flap (in which incisions are made approximately 4–5 mm from the free gingival margin [14]). Furthermore, discordant results were reported due to a wide range of follow-up timings considered, approximately varying from 2 weeks to 4 years after surgical extraction [15].

Regarding daily clinical practice, a greater relevance seems to be usually assigned to early evaluations of periodontal healing after third molar surgery, especially for the second molar site, often disregarding late assessment of tissue conditions. Considering that evidence for the stability of effective wound healing [8,15,16] is essential for a proper comparison between time intervals, the authors thus assumed a follow-up longer than 2 months as notable [12], for it was hypothesized that no strong evidence can be highlighted for or against a specific flap design.

In the light of these considerations, the aim of this retrospective study was to evaluate six-month periodontal healing of lower first and second molar sites, following soft tissue incisions performed to obtain access for surgical extraction of lower third molars (included or affected by dysodontiasis), using a marginal flap with papilla detachment (DETP) or a trapezoidal flap (TRAP). Periodontal healing was evaluated for all indexes related to the stability of soft tissues, primarily producing results in terms of inflammation (BOP) and periodontal attachment (CAL).

## 2. Materials and Methods

### 2.1. Study Sample and Inclusion Criteria

Patients who were consecutively treated for a lower third molar surgery at the Dentistry and Maxillo-facial Surgery Unit (University of Verona) during 2018 were included in a study undertaken as a retrospective evaluation conducted between March 2022 and July 2023 on the available medical records, enabling assessments of satisfaction of the inclusion criteria and with both 1-month and 6-month follow-ups after extraction. Specifically, the inclusion criteria were as follows:

- Patients aged between 14 and 30 years;
- Patients with ASA (American Society of Anesthesiology categorization) category I (any potential organic, physiological, biochemical, or psychological disorders were excluded);
- Written informed consent obtained from each subject involved in the study, and proper forms regularly completed by parents or other related legal guardians for patients under the age of 18 years;
- Pre-surgical assessment of lower third molar level of inclusion and its relationship to the second molar. Third molar positions were categorized using the Pell and Gregory classification (Class I, II, and III according to proximity to the anterior border of the mandibular ramus; class A, B, and C according to proximity to the occlusal plane of the adjacent second molar) [9,17,18,19].

The nature and goals of this study, as well as the anonymity in the scientific use of data, were clearly presented in the consent form. The Declaration of Helsinki and the good clinical practice guidelines for human research were followed during this study’s execution. This retrospective study received approval from the University Institutional Review Board (Protocol “POST-ESTR. LEMBI”, Prog. 3921CESC). This study demonstrated adherence to the STROBE checklist recommendations.

### 2.2. Surgical Techniques

The surgeon performed both pre-surgical evaluations and consequent surgical procedures, as previously described [12]. Each patient underwent a panoramic radiograph and a cone beam computed tomography (CBTC) evaluation [20].

When a patient had bilateral mandibular third molars, they were both extracted during a single procedure. One minute of 0.12% chlorhexidine mouthwash was taken one hour prior to surgery, and one gr of amoxicillin + clavulanic acid was taken orally 24 h prior. Both plexus block and troncular nerve block (articaine 1:100,000 without adrenaline) were performed using local anesthesia (articaine 1:100,000 with adrenaline). To ensure proper wound closure in flap repositing and to allow for initial wound healing, soft tissue incisions had to guarantee a visible section of the epithelial connective tissue and periosteal layers. Additionally, excessive surgical trauma and lacerations of the flap edges had to be avoided. For soft tissue incisions and ensuing subperiosteal dissection, a scalpel blade N.15 (HuFriedy Italy Srl, Milan, Italy) was used.

**Figure 1:**
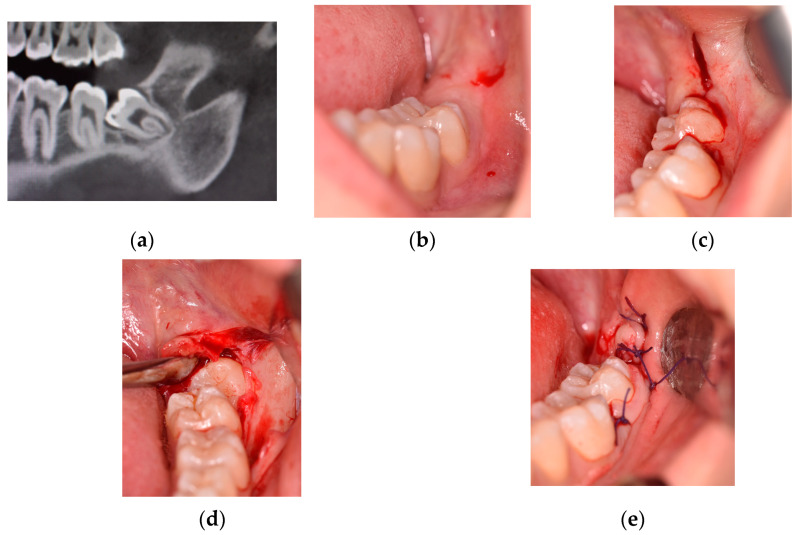
Flap with papilla detachment (class IC position): (**a**) pre-operative detail of cone beam TC evaluation; (**b**) pre-operative clinical evaluation; (**c**) intra-operative distal-releasing incision; (**d**) intra-operative detachment of gingival papilla between the first and second molars; and (**e**) final wound closure.

As shown in Figure 1, the DETP technique [11] can be considered as a modification of an envelope flap, as it employs the periosteal elevator followed by the scalpel. As the incision progresses distally, the gingival papilla between the first and second molars is detached, and the periodontal ligament is also detached from the second molar. The incision begins by inserting the periosteal elevator inside the gingival sulcus along the central-buccal side of the first molar clinical crown. The scalpel was only used for the final releasing incision, which began distally from the distal side of the second molar and moved in a distal-buccal direction and angled at 45 degrees to the dental arch’s ideal prosecution [21].

**Figure 2:**
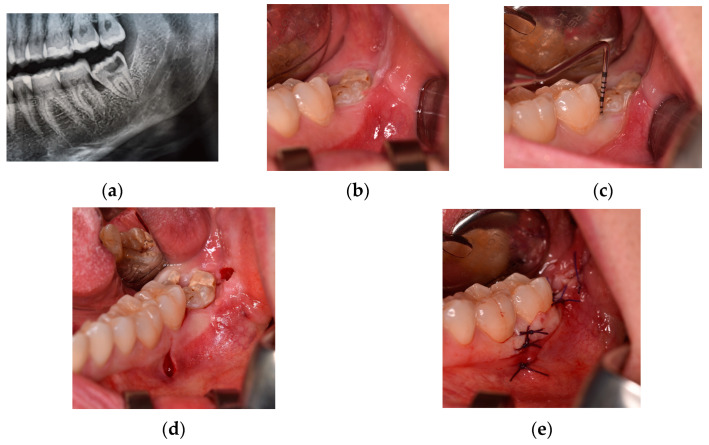
Trapezoidal flap (class IB position): (**a**) pre-operative detail of panoramic radiograph; (**b**) pre-operative clinical evaluation; (**c**) pre-operative periodontal probing of pocket at distal site of the second molar; (**d**) intra-operative mesial- and distal-releasing incisions; and (**e**) final wound closure.

As shown in Figure 2, the TRAP technique [22] provides an intrasulcular-buccal incision at the level of adherent gingiva. It begins at the level of the first molar, then continues at the level of second molar, and then ends with a releasing incision directed towards the distal-buccal side of the second molar, as in DETP. Furthermore, this flap involves a second mesial-releasing incision, which begins at the distal-buccal gingival margin of the first molar, to allow for simpler flap dissection.

Regarding the rest of the surgical procedures, in both cases osteotomy was conducted with appropriate bone-cutting instruments (carbide round bur, rose head bur, chisel, or osteotomy cut); odontotomy was performed with fissure burs; final extractions were performed using the appropriate levers and physics or conventional forceps. Flap closure was finally performed with Vicryl 3.0 and 4.0 sutures (Vycril™ Ethicon, Somerville, NJ, USA).

Post-operative indications and antibiotic prescriptions (875 mg of amoxicillin + 125 mg of clavulanic acid 3 times a day for a week) were given. Patients were checked for suture removal after 7 days, and for follow-up visits after 14 days, one month, and 6 months.

### 2.3. Soft Tissue Assessment

A periodontal probe (Florida Probe; Florida Probes Company, Gainesville, FL, USA) was used to assess periodontal soft tissues [12,23,24] in terms of: PPD (measured in mm as the distance between the gingival margin and the base of the periodontal pocket), CAL (measured in mm as the distance from the CEJ to the location of the probe tip), and VPI (measured as 0 (no plaque) or 1 (plaque), recorded after probing for PPD). In this study, data for BOP (measured as 0 (no bleeding) or 1 (bleeding), recorded after probing for PPD) and REC (measured in mm as the distance between the cementoenamel junction (CEJ) to the gingival margin level) were also available.

Both the first and second molar sites underwent clinical soft tissue examination to measure the average values (expressed in mm, or in percentages for BOP and VPI) of the mesial, central, and distal sites investigated on the buccal/lingual sides. All sites were detected pre-operatively (T0), after 1 month (T1), and after 6 months (T2) and analyzed for each time interval (between T0 and T1, and between T0 and T2).

### 2.4. Statistical Analysis

After creating a database with Microsoft Excel, data analysis was conducted with Stata v.13.0 for Macintosh (StataCorp, College Station, TX, USA). The Shapiro–Wilk test was used to evaluate the normality assumptions for continuous data; means and standard deviations were reported for normally distributed data, with the median and interquartile range (iqr) reported otherwise. Absolute frequencies, percentages, and 95% confidence intervals were reported for categorical data. The comparison between the means of continuous variables in different times was performed using the paired Student’s “*t*” test or Wilcoxon matched-pairs signed-rank test. The comparison of the means among groups was performed using the one-way analysis of variance (ANOVA) or Kruskal–Wallis equality-of-populations rank test. The level of significance was set at 0.05.

## 3. Results

### 3.1. Study Sample

A total of 40 (17 men and 23 women) patients were finally included in the retrospective evaluation, with an equal distribution of patients (20 and 20) for each technique evaluated. The mean age was 22 (14–30) years. The demographics are reported in Table 1.

Regarding flap design (which was performed according to tooth position), DETP was employed in all teeth of class IA and IIA, three teeth of class IIIA, five teeth of class IB, one tooth of class IC, and one tooth of class IIIC; TRAP was used in the rest of the cases.

### 3.2. Soft Tissue Assessment

As during soft tissue examination (involving mesial, central, and distal sites registered on the buccal/lingual sides) no variations at each time interval (between T0 and T1, and between T0 and T2) were found on the lingual sides, the following average values only considered variations on the buccal sides.

#### 3.2.1. Visible Plaque Index

The mean overall values assessed for the first and second molar sites (Table 2 and Table 3) demonstrated moderately increased values between T0 and T1, followed by a slight decrease (for the first molar) and a stable value (for the second molar) up to T2.

For the second molar site (see Table 3), the overall variations at both time intervals were registered as statistically significant (*p* = 0.03 and *p* = 0.02) for a stable mean value of 0.23. Similarly, the TRAP technique registered a statistically significant variation (*p* = 0.02 and *p* = 0.03) at both time intervals for a stable mean value of 0.3. From a clinical point of view, these significant values can be considered as moderately relevant for the second molar site in terms of the VPI.

**Figure 3:**
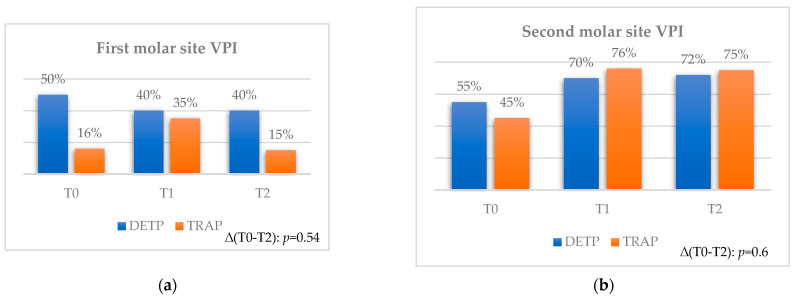
Comparison of the VPI between teeth extracted with TRAP or DETP between T0, T1, and T2; values are presented in %. (**a**) First molar site. (**b**) Second molar site.

No statistical differences were found between the two flaps at each observation time and considering both time intervals (Figure 3a,b).

#### 3.2.2. Bleeding on Probing

The mean overall values assessed for the first and second molar sites (Table 4 and Table 5) demonstrated progressively moderate increased values at each time interval.

For the first molar site (see Table 4), the TRAP technique registered a statistically significant variation (*p* = 0.02 and *p* = 0.01) at both time intervals for mean values of 0.13 and 0.2. For the second molar site (see Table 5), the overall variations at both time intervals were registered as statistically significant (*p* = 0.005 and *p* = 0.001) for mean values of 0.22 and 0.26. Similarly, the DETP technique registered a statistically significant increase (*p* = 0.01) at both time intervals for mean values of 0.28 and 0.36. From a clinical point of view, these significant values for the second molar site can be considered as relevant in terms of BOP.

**Figure 4:**
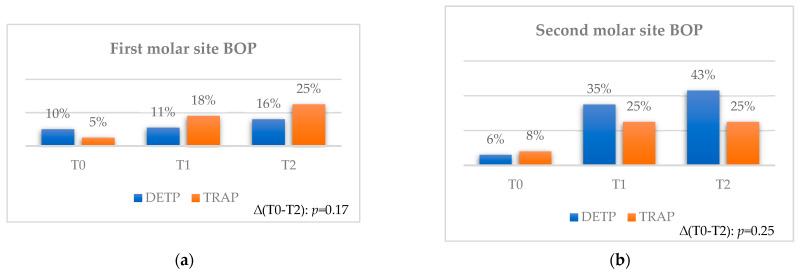
Comparison for BOP between teeth extracted with TRAP or DETP between T0, T1, and T2; values are presented in %. (**a**) First molar site. (**b**) Second molar site.

No statistical differences were found between the two flaps at each observation time and considering both time intervals (Figure 4a,b).

#### 3.2.3. Recession

The mean overall values assessed for the first and second molar sites (Table 6 and Table 7) demonstrated moderately increased values between T0 and T1, followed by a moderate decrease up to T2.

**Figure 5:**
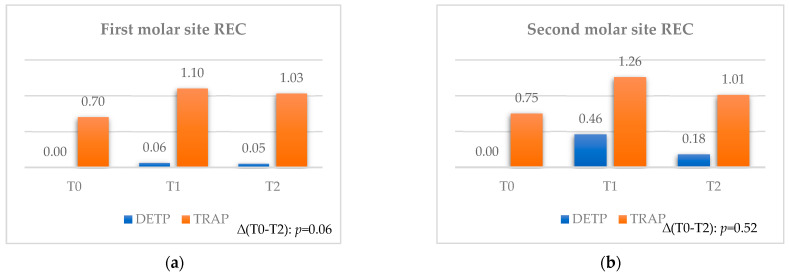
Comparison for REC between teeth extracted with TRAP or DETP between T0, T1, and T2; values are presented in mm. (**a**) First molar site. (**b**) Second molar site.

Furthermore, no statistical differences were found between the two flaps considering the final time interval (Figure 5a,b).

For the first molar site (see Table 6), the overall variations at both time intervals were registered as statistically significant (*p* = 0.004 and *p* = 0.01) for mean values of 0.23 mm and 0.19 mm. Similarly, the TRAP technique registered a statistically significant increase (*p* = 0.01 and *p* = 0.03) at both time intervals for mean values of 0.4 mm and 0.33 mm. From a clinical point of view, only these significant values can be considered as relevant for the first molar site in terms of REC.

For the second molar site (see Table 7), the overall variations at both time intervals were registered as statistically significant (*p* = 0.001 and *p* = 0.003) for mean values of 0.49 mm and 0.21 mm. Similarly, both techniques registered a statistically significant increase (*p* = 0.008 and *p* = 0.003) between T0 and T1, followed by a significant decrease (*p* = 0.04 and *p* = 0.02) up to T2 for final mean values of 0.18 mm and 0.25 mm for DEPT and TRAP, respectively. From a clinical point of view, these significant values can be considered as greatly relevant for the second molar site in terms of REC.

Moreover, eventual significant variations in comparing flaps at each observation time cannot be considered as clinically relevant due to the findings of statistical differences between the two flaps already at T0 (*p* = 0.001 for both the first and second molar sites).

#### 3.2.4. Probing Pocket Depth

The mean values assessed for the first and second molar sites (Table 8 and Table 9) demonstrated evidently increased values between T0 and T1, followed by a moderate decrease up to T2.

**Figure 6:**
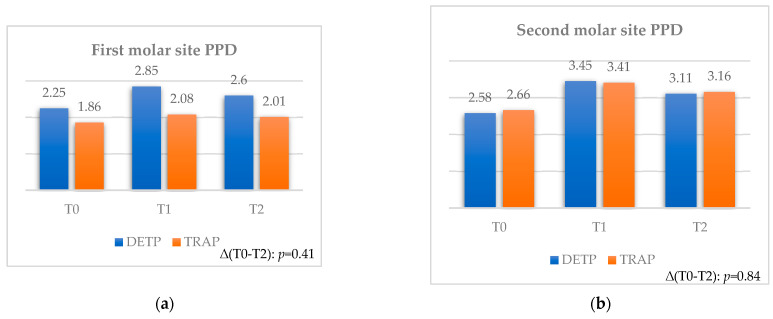
Comparison for PPD between teeth extracted with TRAP or DETP between T0, T1, and T2; values are presented in mm. (**a**) First molar site. (**b**) Second molar site.

Furthermore, no statistical differences were found between the two flaps considering each time interval (Figure 6a,b).

For the first molar site (see Table 8), the overall variations at both time intervals were registered as statistically significant (*p* = 0.001 and *p* = 0.008) for mean values of 0.4 mm and 0.25 mm. Similarly, the DETP technique registered a statistically significant change (*p* = 0.001 and *p* = 0.01) at both time intervals for mean values of 0.6 mm and 0.35 mm. From a clinical point of view, these significant values can be considered as relevant for the first molar site in terms of PPD. Moreover, eventual significant variations for the first molar site in comparing flaps at each observation time cannot be considered as clinically relevant due to the findings of statistical differences between the two flaps already at T0.

For the second molar site (see Table 9), the overall variations at both time intervals were registered as statistically significant (*p* = 0.001) for mean values of 0.8 mm and 0.51 mm. Similarly, both techniques registered a statistically significant increase (*p* = 0.001) between T0 and T1, followed by a significant decrease (*p* = 0.02 and *p* = 0.01) up to T2 for final mean values of 0.53 mm and 0.5 mm for DEPT and TRAP, respectively. From a clinical point of view, these significant values can be considered as greatly relevant for the second molar site in terms of PPD.

#### 3.2.5. Clinical Attachment Loss

The mean values assessed for the first and second molar sites (Table 10 and Table 11) demonstrated moderately increased values between T0 and T1, followed by a moderate decrease up to T2.

**Figure 7:**
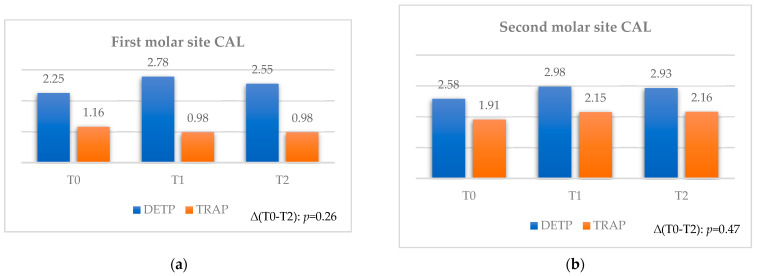
Comparison for CAL between teeth extracted with TRAP or DETP between T0, T1, and T2; values are presented in mm. (**a**) First molar site. (**b**) Second molar site.

Furthermore, no statistical differences were found between the two flaps considering the final time interval (Figure 7a,b).

Statistical differences, both for the first and second molar sites, were found between the two flaps at each observation time. For the first molar site (see Table 10), the DETP technique registered a greater variation (*p* = 0.1) between T0 and T1 compared to TRAP, and a statistically significant increase (*p* = 0.004 and *p* = 0.04) at each time interval, for a mean value of 0.53 mm, decreasing finally to 0.3 mm up to T2. For the second molar site (see Table 11), the DETP technique registered a significantly greater variation (*p* = 0.04) between T0 and T1 compared to TRAP, for a mean value of 0.4 mm, slightly decreasing to 0.35 mm up to T2. From a clinical point of view, these significant values can be considered as relevant for the first and second molar site in terms of CAL. Moreover, eventual significant variations for the second molar site in comparing flaps at each observation time cannot be considered as clinically relevant due to the findings of statistical differences between the two flaps already at T0.

#### 3.2.6. Soft Tissue Assessment According to the Degree of Inclusion

Concerning tooth class, described as the degree of inclusion (see Table 1), no significant differences, both for the overall sample and comparing flap techniques, were assessed for all periodontal indexes between T0 and T2 (see Table 12).

#### 3.2.7. Soft Tissue Assessment According to Patients’ Age

Considering an overall evaluation of soft tissue conditions of the second molar site between T0 and T2 in terms of BOP, PPD, and CAL (see Table 13), no significant differences were assessed for patients 14–25 and >25 years old.

## 4. Discussion

A complete evaluation of post-operative conditions after third molar surgery is fundamental to detect eventual inflammatory complications, such as pain, swelling, trismus, infection, and alveolar osteitis, all implying a relevant negative impact on everyday quality of life of patients [25,26].

As shown by this investigation, which presents a one-month and six-months analysis of data related to the periodontal indexes of PPD, VPI, BOP, REC, PPD, and CAL, regular and proper monitoring of soft tissue conditions [27] can represent an easy and non-invasive measure in disclosing and thus reducing the incidence of the abovementioned complications. Studies in the literature describing methods to avoid possible soft tissue inflammation are heterogeneous [25,26,27,28,29], evidencing the great interest of clinicians in improving patients’ post-operative care.

Concerning the evaluation of the adjacent first and second molar sites, a general increasing trend, between T0 and T1, was assessed for all parameters. Furthermore, a general decreasing trend, up to T2, was assessed for REC, PPD, and CAL; the BOP and VPI indexes showed an increasing trend up to T2 instead.

On the other hand, considering overall variations after 6 months (with the time interval assumed as an effective indicator of stable conditions of soft tissue healing), a moderately increasing tendency was observed between T0 and T2, even if not significant for any of the analyzed parameters. From a clinical point of view, the significant values found can be considered as:

- Moderately relevant for the second molar site in terms of the VPI;
- Relevant for the second molar site in terms of BOP;
- Greatly relevant for the second molar site in terms of REC and PPD;
- Relevant for the first and second molar sites in terms of CAL.

Delving deeper throughout the presented outcomes, BOP and the VPI were not particularly influenced by a specific flap design during the periodontal healing process following the surgical extraction of the lower third molar. Nonetheless, it should be emphasized that an increased percentage of BOP is usually linked to consistent gingival inflammation; therefore, it is characterized by a relevant concentration of cytokines and inflammatory mediators, which certainly do not facilitate tissue repair processes [8,16]. Some studies [30,31] suggested adjuvant therapies (such as photodynamic therapy, hyperbaric oxygen therapy, and topical desiccant agents) to traditional oral hygiene protocols to reduce pain and control bleeding. An increased VPI, indicating greater deposits of bacterial plaques with high proliferation at sites previously subjected to surgical extraction, induces the activation of gingival defensive mechanisms itself, with consequent BOP as a manifestation of inflammation [9,32]. Based on these considerations, and distinctly from the previous two-month follow-up study [12], indexes of gingival inflammation, especially BOP, seem to assume importance for longer follow-up evaluations, as confirmed by other authors [16]. In this regard, BOP was demonstrated to increase more in the first molar site with TRAP, while more in the second molar site with DETP: this can be related to eventual excessive tissue manipulation in the first molar site in the case of the mesial-releasing incision of the TRAP design [28] to allow for easier flap mobilization.

Always regarding TRAP, the authors have assumed that the clinical relevance of greater REC with this technique at the first molar site could be related to the specific design of this flap, which also provides for the release incision at this site, thus interfering with the adherent gingiva and establishing an ideal condition for the development of recession. The two incisions involved in this flap (the intrasulcular incision at the level of the second molar with distal release, and the second release incision from the distal-buccal margin of the first molar with a mesial direction) determine the need for three sites of suture: one at the interdental papilla, between the first and second molars, and two sutures at the level of the second-releasing incision [33]. An area of ischemia could therefore be created in the space between the first and the other two sutures, with a reduced vascular supply: this matter could constitute the biological justification for the potential development of a recession, localized at the vestibular level of the first molar, as an expression of tissues suffering during the healing phase [34]. As seen from our experience, the TRAP technique provides the formation of a gingival recession at also the second molar site. Moreover, even performing a flap with the detachment of the papilla, the second molar site appears to be subjected to gingival recession, as this element represents in both cases the site for the incision aimed to surgical access [32,35].

Concerning PPD, according to studies in the literature, the choice of a specific flap design does not seem to generally influence clinical outcomes in limiting the increase in probing for adjacent elements in a mid-term follow-up [22,36,37]. An increase in PPD for both flap designs at the first and second molar sites represents a trend related to a limited follow-up period of only one month (first time interval), with it being known that at least three months of follow-up [7] would be necessary to evaluate a complete healing process. In this study, the increasing pattern of PPD is compatible with an early post-surgery period [13], followed by its slight decrease or stabilization after 6 months, as demonstrated by other investigations with equal or longer follow-ups and different flap designs [8,15,16], in some cases even with data at one-year after surgery [16]. Moreover, current systematic reviews [11,15] have found that post-operative PPD, continuously decreasing over time, can be found as lower compared to the baseline in cases of follow-ups longer than 3 months.

Data reported for CAL revealed overall favorable conditions, both for the first and second molar sites, in patients treated with TRAP compared to DETP. Combining these outcomes with the data for REC, mainly worse for TRAP, and PPD results, instead mainly worse for DETP, it can be seen how both time intervals (1 month and 6 months) were necessary to highlight the final stability of soft tissue conditions for both techniques through measurements of CAL.

A secondary aspect evaluated in this study was the response of soft tissues between T0 and T2, also according to different pre-operative degrees of inclusion, for the overall sample and comparing techniques: significant differences between groups were not shown by any of the indexes apart from the technique used, as already found in another study by the same research group [12]. The outcomes appeared to be heterogeneous, considering the influence of tooth position on post-operative periodontal healing depending on the flap design chosen [3,38], underlining the progressive level of difficulty from class A to B and C [5,7], or, on the other hand, the unreliability of the Pell–Gregory classification in predicting a difficult procedure [38,39]. As a variety of traditional classifications considered by authors were recently enriched with further details from 3D evaluations of tooth position [3], a complete analysis of periodontal indexes according to the degree of inclusion, still lacking in the literature, encourages further investigations.

In addition, regarding the entity of surgical ostectomy based on the available bone density, no significant differences were found in terms of the BOP, PPD, and CAL of the second molar site between patients 14–25 and >25 years old, evidencing a limited impact of this aspect on six-month soft tissue stability. Despite an age above 25 years having being reported to be associated with a higher number of complications in third molar surgery [40], 75% of patients in this study were younger. Under this proposal, the small sample evaluated represents a strong limitation for this study.

Finally, while a previously published study by the same research group [12] evaluated a non-homogenous group of 80 patients with a two-month follow-up and treated with four surgical techniques, this study presented a smaller, but more homogeneous, sample of 40 patients followed for one month and six months after surgical treatment with two techniques to compare both the earliest and mid-term conditions of wound healing. In this regard, a complete evaluation of periodontal parameters, a homogeneous distribution of flap design between groups, and a follow-up of 6 months all constitute, on the other hand, possible strengths for the present study.

## 5. Conclusions

The present study revealed overall stable outcomes of soft tissue conditions after third molar extraction in terms of inflammation and periodontal attachment, apart from the flap performed and the starting tooth degree of inclusion. Even if mostly present in the patients treated with TRAP (for the reduced vascular supply occurring in the ischemic area with sutures, not usually assessed in subjects treated with DETP), the formation of a gingival recession for both elements adjacent to the element extracted does not seem to depend on the chosen flap.

Regarding the increase in PPD in the early post-surgical follow-up, the use of a specific flap design does not seem to be particularly important up to six months. We are aware, however, of the need for even longer follow-ups to properly assess periodontal healing.

## Figures and Tables

**Table 1:** Patients and tooth characteristics at baseline; values are presented in *n* (%).

Variable	*n*	%
Sex		
Male	17	42.50
Female	23	57.50
Age		
14–25 years	30	75.00
25–30 years	10	25.00
Tooth site		
Element 38	22	55.00
Element 48	18	45.00
Surgical technique		
Trapezoidal flap (TRAP)	20	50.00
Papilla detachment (DETP)	20	50.00
Tooth class (Pell and Gregory classification)		
IA	4	10.00
IIA	6	15.00
IIIA	7	17.50
IB	7	17.50
IIB	3	7.50
IIIB	7	17.50
IC	1	2.50
IIC	2	5.00
IIIC	3	7.50

**Table 2:** Assessment of the VPI for the first molar site in teeth extracted with TRAP (trapezoidal flap) or DETP between T0 and T2; values are presented in %.

		T0	T1	T2	∆(T0-T1)	*p*-Value	∆(T0-T2)	*p*-Value
	Overall	0.33 (0.52)	0.37 (0.66)	0.27 (0.51)	0.04 (0.66)	0.96	(-)0.05 (0.54)	0.52
Technique								
	DETP	0.50 (0.60)	0.40 (0.68)	0.4 (0.6)	(-)0.1 (0.78)	0.38	(-)0.1 (0.72)	0.53
	TRAP	0.16 (0.36)	0.35 (0.67)	0.15 (0.36)	0.18 (0.50)	0.08	(-)0.01 (0.27)	0.97
	*p*-value	0.06	0.75	0.13	0.09		0.54	

**Table 3:** Assessment of the VPI for the second molar site in teeth extracted with TRAP or DETP between T0 and T2; values are presented in %.

		T0	T1	T2	∆(T0-T1)	*p*-Value	∆(T0-T2)	*p*-Value
	Overall	0.50 (0.67)	0.73 (0.82)	0.73 (0.81)	0.23 (0.65)	0.03 *	0.23 (0.64)	0.02 *
Technique								
	DETP	0.55 (0.60)	0.70 (0.73)	0.72 (0.71)	0.15 (0.67)	0.38	0.16 (0.66)	0.27
	TRAP	0.45 (0.75)	0.76 (0.91)	0.75 (0.91)	0.31 (0.65)	0.02 *	0.3 (0.63)	0.03 *
	*p*-value	0.38	0.91	0.87	0.38		0.6	

* Statistically significant differences between groups/observation times.

**Table 4:** Assessment of BOP for the first molar site in teeth extracted with TRAP or DETP between T0 and T2; values are presented in %.

		T0	T1	T2	∆(T0-T1)	*p*-Value	∆(T0-T2)	*p*-Value
	Overall	0.07 (0.20)	0.15 (0.33)	0.2 (0.34)	0.07 (0.35)	0.21	0.13 (0.35)	0.3
Technique								
	DETP	0.10 (0.24)	0.11 (0.31)	0.16 (0.33)	0.01 (0.42)	0.79	0.06 (0.45)	0.65
	TRAP	0.05 (0.16)	0.18 (0.36)	0.25 (0.38)	0.13 (0.27)	0.02 *	0.2 (0.31)	0.01 *
	*p*-value	0.39	0.44	0.47	0.11		0.17	

* Statistically significant differences between groups/observation times.

**Table 5:** Assessment of BOP for the second molar site in teeth extracted with TRAP or DETP between T0 and T2; values are presented in %.

		T0	T1	T2	∆(T0-T1)	*p*-Value	∆(T0-T2)	*p*-Value
	Overall	0.07 (0.20)	0.30 (0.41)	0.34 (0.4)	0.22 (0.47)	0.005 *	0.26 (0.49)	0.001 *
Technique								
	DETP	0.06 (0.13)	0.35 (0.42)	0.43 (0.43)	0.28 (0.42)	0.01 *	0.36 (0.48)	0.01 *
	TRAP	0.08 (0.26)	0.25 (0.41)	0.25 (0.35)	0.16 (0.53)	0.15	0.16 (0.49)	0.08
	*p*-value	0.48	0.30	0.15	0.42		0.25	

* Statistically significant differences between groups/observation times.

**Table 6:** Assessment of REC for the first molar site in teeth extracted with TRAP or DETP between T0 and T2; values are presented in mm as median (iqr, interquartile range).

		T0	T1	T2	∆(T0-T1)	*p*-Value	∆(T0-T2)	*p*-Value
	Overall	0.35 (0.78)	0.58 (0.93)	0.54 (0.94)	0.23 (0.56)	0.004 *	0.19 (0.55)	0.01 *
Technique								
	DETP	0.00 (0.00)	0.06 (0.23)	0.05 (0.22)	0.06 (0.23)	0.15	0.05 (0.22)	0.31
	TRAP	0.70 (1.00)	1.10 (1.08)	1.03 (1.12)	0.40 (0.73)	0.01 *	0.33 (0.73)	0.03 *
	*p*-value	0.001 *	0.001 *	0.001 *	0.03 *		0.06	

* Statistically significant differences between groups/observation times.

**Table 7:** Assessment of REC for the second molar site in teeth extracted with TRAP or DETP between T0 and T2; values are presented in mm as median (iqr, interquartile range).

		T0	T1	T2	∆(T0-T1)	*p*-Value	∆(T0-T2)	*p*-Value
	Overall	0.37 (0.83)	0.86 (0.89)	0.59 (0.87)	0.49 (0.65)	0.001 *	0.21 (0.42)	0.003 *
Technique								
	DETP	0.00 (0.00)	0.46 (0.70)	0.18 (0.41)	0.46 (0.70)	0.008 *	0.18 (0.41)	0.04 *
	TRAP	0.75 (1.05)	1.26 (0.90)	1.01 (1.02)	0.51 (0.62)	0.003 *	0.25 (0.44)	0.02 *
	*p*-value	0.001 *	0.003 *	0.004 *	0.59		0.52	

* Statistically significant differences between groups/observation times.

**Table 8:** Assessment of PPD for first molar site in teeth extracted with TRAP or DETP between T0 and T2; values are presented in mm as median (iqr, interquartile range).

		T0	T1	T2	∆(T0-T1)	*p*-Value	∆(T0-T2)	*p*-Value
	Overall	2.05 (0.47)	2.46 (0.68)	2.3 (0.58)	0.40 (0.61)	0.001 *	0.25 (0.54)	0.008 *
Technique								
	DETP	2.25 (0.38)	2.85 (0.43)	2.6 (0.49)	0.60 (0.58)	0.001 *	0.35 (0.54)	0.01 *
	TRAP	1.86 (0.48)	2.08 (0.68)	2.01 (0.53)	0.21 (0.58)	0.08	0.15 (0.55)	0.17
	*p*-value	0.006 *	0.001 *	0.001 *	0.10		0.41	

* Statistically significant differences between groups/observation times.

**Table 9:** Assessment of PPD for the second molar site in teeth extracted with TRAP or DETP between T0 and T2; values are presented in mm as median (iqr, interquartile range).

		T0	T1	T2	∆(T0-T1)	*p*-Value	∆(T0-T2)	*p*-Value
	Overall	2.62 (0.63)	3.43 (0.74)	3.14 (0.77)	0.80 (0.71)	0.001 *	0.51 (0.83)	0.001 *
Technique								
	DETP	2.58 (0.58)	3.45 (0.75)	3.11 (0.75)	0.86 (0.77)	0.001 *	0.53 (1.01)	0.02 *
	TRAP	2.66 (0.69)	3.41 (0.76)	3.16 (0.82)	0.75 (0.66)	0.001 *	0.5 (0.62)	0.01 *
	*p*-value	0.86	0.93	0.92	0.67		0.84	

* Statistically significant differences between groups/observation times.

**Table 10:** Assessment of CAL for the first molar site in teeth extracted with TRAP or DETP between T0 and T2; values are presented in mm as median (iqr, interquartile range).

		T0	T1	T2	∆(T0-T1)	*p*-Value	∆(T0-T2)	*p*-Value
	Overall	1.70 (1.13)	1.88 (1.45)	1.76 (1.33)	0.17 (0.99)	0.07	0.05 (0.91)	0.25
Technique								
	DETP	2.25 (0.38)	2.78 (0.54)	2.55 (0.56)	0.53 (0.64)	0.004 *	0.3 (0.59)	0.04 *
	TRAP	1.16 (1.36)	0.98 (1.52)	0.98 (1.43)	(-)0.18 (1.16)	0.66	(-)0.18 (1.12)	0.76
	*p*-value	0.002 *	0.001 *	0.001 *	0.10		0.26	

* Statistically significant differences between groups/observation times.

**Table 11:** Assessment of CAL for the second molar site in teeth extracted with TRAP or DETP between T0 and T2; values are presented in mm as median (iqr, interquartile range).

		T0	T1	T2	∆(T0-T1)	*p*-Value	∆(T0-T2)	*p*-Value
	Overall	2.25 (0.95)	2.56 (1.31)	2.55 (1.08)	0.31 (0.97)	0.28	0.3 (0.86)	0.06
Technique								
	DETP	2.58 (0.58)	2.98 (1.22)	2.93 (0.73)	0.40 (1.15)	0.17	0.35 (0.98)	0.08
	TRAP	1.91 (1.14)	2.15 (1.30)	2.16 (1.25)	0.23 (0.77)	0.18	0.25 (0.74)	0.15
	*p*-value	0.01 *	0.02 *	0.02 *	0.04 *		0.47	

* Statistically significant differences between groups/observation times.

**Table 12:** Analysis of soft tissue indexes of periodontal healing between T0 and T2, according to the pre-operative degree of inclusion, for the overall sample and comparing different flap techniques; *p*-values are reported.

Degree of Inclusion (IA, IIA, IIIA, IB, IIB, IIIB, IC, IIC, and IIIC)		Overall	Technique	
			DETP	TRAP
First molar site VPI ∆(T0-T2)	*p*-value	0.06	0.57	0.27
Second molar site VPI ∆(T0-T2)	*p*-value	0.63	0.09	0.54
First molar site BOP ∆(T0-T2)	*p*-value	0.23	0.18	0.08
Second molar site BOP ∆(T0-T2)	*p*-value	0.07	0.73	0.99
First molar site REC ∆(T0-T2)	*p*-value	0.14	0.51	0.35
Second molar site REC ∆(T0-T2)	*p*-value	0.98	0.83	0.16
First molar site PPD ∆(T0-T2)	*p*-value	0.39	0.78	0.47
Second molar site PPD ∆(T0-T2)	*p*-value	0.45	0.07	0.73
First molar site CAL ∆(T0-T2)	*p*-value	0.82	0.34	0.09
Second molar site CAL ∆(T0-T2)	*p*-value	0.12	0.92	0.53

**Table 13:** Analysis of soft tissue conditions in terms of BOP, PPD, and CAL between T0 and T2, according to age intervals (14–25 years and 25–30 years), for the overall sample and comparing different flap techniques; *p*-values are reported.

Second Molar Site	Age		
	14–25 Years	25–30 Years	*p*-Value
BOP ∆(T0-T2)	0.32 (0.86)	0.1 (0.53)	0.56
PPD ∆(T0-T2)	0.50 (0.92)	0.57 (0.26)	0.98
CAL ∆(T0-T2)	0.28 (0.45)	0.37 (0.02)	0.08

## Data Availability

The data presented in this study are available on request from the corresponding author.
